# Cultural and migration-related aspects of trauma-related disorders

**DOI:** 10.1192/j.eurpsy.2025.51

**Published:** 2025-08-26

**Authors:** M. Schouler-Ocak

**Affiliations:** Psychiatry and Psychotherapy, Charité - Universitätsmedizin Berlin, Berlin, Germany

## Abstract

**Abstract:**

Culture fundamentally influences human thinking, feeling and behaviour. The integration of cultural contexts into psychotherapeutic treatment is therefore essential, especially in light of the increasing diversity of our society. In addition, psychological stress following experiences of migration or flight requires special treatment expertise. Thus, intercultural psychotherapy is an essential approach in addressing mental health issues across diverse populations.

Life-threatening events, to which we feel helpless and at the mercy of, can inflict severe psychological trauma on us. If the event is too aversive, too horrific, and therefore cannot be autobiographically interwoven, fragments of memory are created that are not modified over time - like ordinary memories - but remain rigid, as if **‘**frozen’. They are activated by triggers reminiscent of the original situation and then reappear, e.g. in the form of flashbacks. The high number of people with migration and refugee backgrounds with trauma-induced secondary disorders calls for culturally sensitive, trauma-focussed psychotherapies to close the enormous gap in care. Numerous psychotherapies are available for the treatment of trauma-related disorders, some of them have yet to be evaluated - or modified for the treatment of traumatised persons in the context of migration and flight in the case of those that have already been well evaluated. This presentation will give an overview on cultural and migration-related aspects of trauma-related disorders.

**Disclosure of Interest:**

None Declared

